# Longitudinal Daily Living Limitations and Cognitive Status: Results from the 1998-2016 Health and Retirement Study

**DOI:** 10.1093/geroni/igaa057.3306

**Published:** 2020-12-16

**Authors:** Benson Wu, Mohammad Usama Toseef, Wassim Tarraf, Ariana Stickel, Sonya Kaur, Alberto Ramos, Hector Gonzalez

**Affiliations:** 1 University of California, San Diego School of Medicine, La Jolla, California, United States; 2 Wayne State University, Detroit, Michigan, United States; 3 Shiley-Marcos Alzheimer’s Disease Research Center, La Jolla, California, United States; 4 University of Miami Miller School of Medicine, Miami, Florida, United States

## Abstract

Data increasingly points to midlife health and modifiable risk factors as critical targets for improving older-age health outcomes and mitigating potential cognitive impairment and disease. We used biennial Health and Retirement Study data (1998-2016) collected on adults ages 50-64 years who did not meet criteria for dementia at baseline and who remained living by 2016 (unweighted-n=4,803). Cognitive status was defined using Langa-Weir criteria: Normal, Cognitively Impaired Not Dementia (CIND), and Dementia. We examined how 18-year patterns in activities of daily living (ADLs) and instrumental activities of daily living (IADLs) predicted cognitive status in 2016. We used latent class analysis to extract longitudinal phenotypes of activities limitations, followed by survey multinomial logistic regressions to examine their associations with cognitive status and test for race/ethnic modifications. We identified three groups of functional impairment: (1) gradually increasing (15.7%), (2) stable elevated (5.6%), and (3) minimal dysfunction (78.7%). After covariates adjustment, both the gradual and stable elevated impairment groups (vs. minimal) had substantially higher relative risk ratios (RRR) for dementia (RRR=5.71[3.89;8.39] and RRR=7.87[4.23,14.64]) and CIND (RRR=2.21 [1.69,2.88] and RRR=1.92[1.16;3.17]). We detected modifications by race/ethnicity such that Hispanics with stable elevated impairment had a higher probability of dementia compared to their White counterparts. The results varied for Blacks and did not significantly differ from Whites. Data-driven methods may improve our understanding of heterogeneous functional impairment patterns among late middle-aged adults and allow for tailored ADRD prevention strategies. Focused risk-based interventions can yield important public health savings and reductions in structural, social, and individual health burdens.

